# Prevalence and predictive factors for renouncing medical care in poor populations of Cayenne, French Guiana

**DOI:** 10.1186/s12913-016-1284-y

**Published:** 2016-01-28

**Authors:** Larissa Valmy, Barbara Gontier, Marie Claire Parriault, Astrid Van Melle, Thomas Pavlovsky, Célia Basurko, Claire Grenier, Maylis Douine, Antoine Adenis, Mathieu Nacher

**Affiliations:** 1 Centre d’Investigation Clinique Antilles-Guyane, Inserm 1424, Centre Hospitalier Andrée Rosemon, Av. des Flamboyants, 97306 Cayenne cedex, France; 2 Emergency Department, Centre Hospitalier Andrée Rosemon, Av. des Flamboyants, 97306 Cayenne cedex, France; 3 Croix Rouge Française, Centre de Prévention Santé, 25 rue Docteur Roland Barrat, 97300 Cayenne, France

**Keywords:** Health care, Renouncement, Poor populations, Logistic regression, Tree analysis, French Guiana

## Abstract

**Background:**

Access to health care is a global public problem. In French Guiana, there exists social inequalities which are specially marked amongst immigrants who make up a third of the population. Health care inequalities are prevalent. The objective of this study was to determine factors associated with why health care amongst the poor population of Cayenne was renounced.

The study was cross sectional. It focused on knowledge, attitudes, practices and beliefs of the population living in poor neighborhoods of the Cayenne area.

**Methods:**

Populations coming at the Red Cross mobile screening unit in poor urban areas of Cayenne were surveyed from July 2013 to June 2014. Structured questionnaires consisted of 93 questions. Written informed consent was requested at the beginning of the questionnaire. The predictors for renouncing medical care were determined using logistic regression models and tree analysis.

**Results:**

Twenty percent of persons had renounced care. Logistic regression showed that renouncement of health care was negatively associated with having no regular physician Adjusted Odds Ratio (AOR) = 0.43 (95 % CI = 0.24–0.79) and positively associated with being embarrassed to ask certain questions AOR = 6.81 (95 % CI = 3.98–11.65) and having been previously refused health care by a doctor AOR = 3.08 (95 % CI = 1.43–6.65). Tree analysis also showed that three of these variables were linked to renouncement, with feeling shy to ask certain questions as the first branching.

**Conclusion:**

Although most people felt it was easy to see a doctor, one in five had renounced health care. The variables identified by the models suggest vulnerable persons generally had previous negative encounters with the health system and felt unwanted or non eligible for healthcare. Health care mediation and welcoming staff may be simple solutions to the above problems which were underscored in our observations.

## Background

Health inequalities between countries and within countries remain salient [1]. Both absolute and relative material standards matter, with the later having a greater importance [2, 3]. Inequalities in income are paralleled by inequalities in health [4]. Richer countries generally have better indicators than poorer countries, however, there is great heterogeneity [5]. French Guiana has the highest GDP per capita on the South American continent, attracting numerous immigrants in search of a better life. Thus, a third of the population are immigrants, a great proportion of whom have no residence permit [6]. The health system of French Guiana is French with modern facilities and the possibility of obtaining universal health insurance, including for illegal foreign citizens residing for more than 3 months on the territory. As for all French territories, mitigating health inequalities remains a major public health goal. There are great socio-sanitary differences between the various French regions, notably for French Guiana [7]. Previous studies in French Guiana have shown that undocumented immigrants reported a poorer health status [8]. Studies have also shown that immigrants were tested later for HIV [9], were more likely to interrupt follow up [10], and, for cancer, had more advanced stages upon diagnosis [11]. The complexities of a poor immigrants’ life, of the health care system and of the administrative requirements to access to rights, the lack of transport, and the refusal by some private practitioners to see patients with universal health insurance are all potential determinants of a sick patient to renounce to see a doctor [12].

We thus conducted a study in patients consulting the Red Cross screening truck in impoverished neighborhoods in Cayenne to determine how frequently persons had renounced healthcare and the variables that were most frequently associated with this.

## Methods

### Ethical aspects

The study protocol was approved by the Comité d’Evaluation Ethique de l’Inserm (CEEI/IRB) (n°13–107) and the Commission Nationale Informatique et Liberté (CNIL) (authorisation n°1680353v0).

### Study design

The study was cross-sectional. It was a descriptive and comparative knowledge, attitudes, practices, and beliefs study.

### Setting

The study was conducted from from July 1st, 2013 through June 30th, 2014 in poor neighborhoods of the Cayenne area. Neighborhoods were defined as « poor » if one of the following criteria were verified: neighborhood figuring in the City Hall list of « Contrats Urbains de Cohésion Sociale » (CUCS, urban contracts of social cohesion); spontaneous habitat zone (housing without building permits); neighborhoods without running water and/or electricity and/or municipal handling of waste and garbage.

### Participants

The study population was poor populations. The surveyed population was adults, seeking free screening services at the Red Cross mobile screening unit (truck offering testing for HIV, diabetes, high blood pressure).

The surveyed areas are sensibly different from the rest of Cayenne and selected for this. They are mostly, but not exclusively inhabited, by immigrants. Given the small size of Cayenne, the surveyed areas are not very far from the health structures. However they are devoid of health structures, social facilities, and public transportation is often not available. The estimated population living in the surveyed areas was 46 000 relative to the 101 412 persons living in the greater Cayenne area.

### Outcome criteria

The renouncement of care which was defined by answering yes to the question “Have you been ill or had pains and renounced care?”. The answer to this question had no time frame but referred to French Guiana and thus represents a prevalence during the stay in French Guiana.

### Inclusion criteria

Adults, over 18 years of age.

### Non inclusion criteria

Person belonging to the study population refusing to participate, having previously participated, <18 years of age.

### Sampling method

Samples were stratified by inclusion centers, with an equivalent proportion of persons relative to the estimated source population seen at each site.

The Red Cross mobile unit exists since 2012. It allows going towards populations in the poorest neighborhoods. Nurses are accompanied by health mediators from the local communities (DAAC, development, accompaniment, animation, cooperation). Twice a month a doctor is there to check vaccinations.

### Data collection

The trained multilingual surveyors from the targeted communities asked all persons consulting the mobile unit. Written and oral information was given to all eligible persons. Written informed consent was requested at the beginning of the questionnaire. Persons accepting to participate to the study were interviewed face to face in their native language using a structured questionnaire that had been translated in local languages (Haitian Creole, Spanish, Portuguese, English, SrananTongo). The study was proposed to the first person arriving on site and then the second questionnaire was proposed when the surveyor had finished with the first person, and so on until the work day was finished.

Questionnaires were anonymous and numbered for each study site and according to their rank in passage that day. The questionnaires had 93 questions divided in five parts: sociodemographic profile, health profile, health practices, attitudes, practices and knowledge on health matters and health insurance. The EPICES score was calculated as described elsewhere [13]. The interview lasted up to 60 min. The initial questionnaire was devised by the Clinical Investigation Center and was reviewed and modified by the partners (Health insurance, municipalities NGOs Red Cross, Médecins du Monde, DAAC). It was pretested on ten persons of the target population to optimize its comprehension and the perception.

Patients were compensated for the time they gave the interviewer and were given a 5 euro voucher plus prevention material on diabetes, high blood pressure, HIV, and information about access to health insurance. Questionnaires were reviewed by the study coordinator, who then validated them if they were coherent.

### Statistical methods

Data analysis was performed using R. Univariate analysis was performed then frequency distributions for each covariate were compared between those who had renounced health care and those that had not. Thematic logistic regression models considering significant variables (*p* < 0.05) were computed for different set of questions: sociodemographic profile, health profile, medical practices, attitudes, attitudes, practices and knowledge on health matters and health insurance. Finally, the variables selected from these thematic multivariate models were added to a global logistic regression model. The goodness of fit test was used. The most parsimonious models were selected using Akaike’s information criterion.

In addition CART analyses were performed using R [14, 15]. Classification And Regression Tree analysis is a non parametric multidimensional exploratory method. It is a segmentation method based on the construction of a decision tree. It aims to partition the sample using explanatory variables so that the obtained segments are as homogenous as possible regarding the dependent variable. It proceeds by successive iterations, the sample first being partitioned in homogenous sub-samples, each of which is then partitioned in homogenous sub samples, iteratively. In some cases, the trees obtained being too intricate, pruning is used to obtain a more parsimonious model.

## Results

**Fig. 1:**
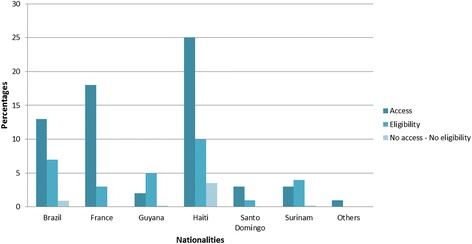
Descriptive analysis. Description of the study population according to nationalities, access, eligibility and no eligibility to healthcare system - Results expressed as percentage - Others : Guinea Bissau, Dominica and Peru

Between July 2013 and June 2014, a total of 546 questionnaires were filled. The refusal rate was zero. Table 1 provides a description of the study population stratified into two groups according healthcare renouncement. This table considers significant variables in the different sets of questions and other variables as age, gender, social vulnerability, income and perception of how easy it is to see a doctor. Table 2 presents the variables associated with healthcare renouncement and the p values. There were 51.8 % of women. The average age was 37.0 (sd = 12.9) in women and 35.6 (sd = 13.7) in men. According to the EPICES score using the 30 threshold, 477 (87.4 %) were socially precarious. Most of these persons came from foreign countries, 47 % were foreign having some form of health insurance and additional 27 % were foreign without insurance but were eligible to it as shown in Fig. 1. Overall, 5 % were foreigners not eligible for health insurance.

**Table 1:** Descriptive analysis. Description of the study population stratified according to renouncement – Results expressed as numbers (%) or medians and interquartile ranges (25–75 %)

	Renouncement (n)	No renouncement (n)
Sociodemographic profile		
Age	33 (26–42)	35 (25–45)
Gender		
Female	52 (46)	231 (53)
Male	61 (54)	202 (47)
Being Haitian		
No	57 (50)	280 (65)
Yes	56 (50)	153 (35)
Being in France, less than a year:		
No	78 (69)	298 (69)
Yes	20 (18)	42 (10)
Non applicable	15 (13)	93 (21)
Marital status		
In a couple/family/roomate	38 (34)	196 (45)
Single	75 (66)	237 (55)
Social vulnerability		
No	5 (4)	64 (15)
Yes	108 (96)	369 (85)
Income level		
Less than net minimum wage	89 (79)	294 (68)
Around net minimum wage	12 (10)	90 (21)
More than net minimum wage	11 (10)	46 (10)
No response	1 (1)	3 (1)
Having financial difficulties		
No	28 (25)	202 (47)
Yes	82 (73)	229 (53)
Having financial help		
No	53 (47)	253 (58)
Yes	59 (52)	178 (41)
Taking Holidays		
No	92 (81)	310 (72)
Yes	21 (19)	123 (28)
Reason for coming		
Health insurance	94 (83)	408 (94)
Other	19 (17)	25 (6)
Being shy to ask certain questions or to go through medical or administrative appointments		
No	50 (44)	381 (88)
Yes	63 (56)	49 (11)
Health profile		
Health status 3 years ago:		
The same	43 (38)	127 (29)
Better	52 (46)	268 (62)
Worse	18 (16)	37 (9)
Having received dental care in the past 3 years		
No	27 (24)	181 (42)
Yes	86 (76)	250 (58)
Having good sight		
No	28 (25)	70 (16)
Yes	85 (75)	363 (84)
Medical practice, attitudes		
Having consulted the emergency ward in the past year		
No	47 (42)	95 (22)
Yes	66 (58)	338 (78)
Having a family physician		
No	64 (57)	133 (31)
Yes	49 (43)	300 (69)
Thinking it is easy to see a doctor		
No	11 (10)	22 (5)
Yes	75 (66)	328 (76)
No response	27 (24)	83 (19)
Having time to explain problems when you see a doctor		
Yes	90 (80)	379 (88)
No	19 (17)	32 (7)
Never consulted	4 (3)	22 (5)
Having been refused health care by a doctor in French Guiana		
No	87 (77)	387 (89)
Yes	24 (21)	25 (5)
Never consulted	1 (2)	20 (6)
Having been refused health care by the PASS		
No	48 (42)	164 (38)
Yes	11 (10)	15 (3)
Never consulted	53 (47)	254 (59)
Attitudes, practices and knowledge on health matters		
Knowing about complementary health insurance		
Yes	51 (45)	264 (61)
No	61 (54)	160 (37)
Knowing the difference between CMU and AME		
Yes	48 (42)	233 (54)
No	64 (57)	191 (44)
Thinking need to feel pain or malaise to be sick		
No	71 (32)	325 (75)
Yes	36 (63)	93 (22)
Knowing infrastructures or associations which help people in administrative process		
Yes	70 (62)	326 (75)
No	43 (38)	106 (25)
HIV-knowledge Quizz		
< 7 true responses	74 (65)	226 (52)
7true responses	39 (35)	207 (48)
Health insurance		
Having documentation proving health insurance		
No	53 (47)	137 (32)
Yes	60 (53)	296 (68)
Understanding mail from the health insurance		
Yes	74 (65)	337 (78)
No	9 (8)	11 (2)
Never received	30 (27)	85 (20)

**Table 2:** Bivariate analysis. Variables associated with a history of healthcare renouncement

	*p*
Sociodemographic profile	
Being Haitian	0.01
Being in France, less than a year	0.02
Social vulnerability	0.005
Marital status	0.03
Having financial difficulties	<0.001
Having financial help	0.04
Taking Holidays	0.05
Reason for coming	<0.001
Being shy to ask certain questions or to go through medical or administrative appointments	<0.001
Health profile	
Health status 3 years ago	0.004
Having received dental care in the past 3 years	<0.001
Having good sight	0.05
Medical practice, attitudes	
Having consulted the emergency ward in the past year	<0.001
Having a family physician	<0.001
Having time to explain problems when you see a doctor	0.01
Having been refused health care by a doctor in French Guiana	<0.001
Having been refused health care by the PASS	0.008
Attitudes, practices and knowledge on health matters	
Knowing about complementary insurance	0.004
Knowing about the difference between CMU and AME	0.05
Thinking one needs to feel pain or malaise to be sick	0.04
Knowing about infrastructures or associations which help people in administrative process	0.01
HIV-knowledge Quizz	0.02
Health insurance	
Having documentation proving health insurance	0.003
Understanding mail from the health insurance	0.004

A significant proportion of persons had very low incomes for the standard of life of French Guiana (medical consultation costs: 23 Euro; net minimum wage: 1 137 Euros): 20 % of patients earned less than 300 Euros per month, 35.2 % less than 500 Euros, and 55 % less than 800 Euros per month.

Overall, 113 persons (20.7 %) had previously renounced health care whereas 433 had never done so (79.3 %). Renouncement of care was more frequent in the precarious group (*p* = 0.005).

Four hundred and three persons (73.8 %) thought it was easy to see a doctor in Cayenne. Among those that had never renounced care 75.8 % though it was easy to see a doctor. Among those that had previously renounced for care still 66.4 % thought it was easy to see a doctor.

For those having arrived in French Guiana for less than a year, 20 (18 %) had renounced whereas 78 (69 %) of those having arrived before 2013 had renounced, *p* = 0.02 (see Table 2).

Table 3 summarizes the differences between the two groups for different variables and shows adjusted odds ratios for thematic logistic regression models. For the sociodemographic variables, financial difficulties and being shy to ask certain questions or to go through medical or administrative appointments were significantly associated with the outcome. For the health profile, only the fact of having received dental care in the past 3 years was significant. For the medical practice and attitudes variables, having consulted the emergency ward in the past year, having a family physician and having been refused care by a doctor were significant predictors. For the variables pertaining to health insurance, having health insurance and understanding mail from health insurance were predictors.

**Table 3:** Thematic multivariate analysis. Variables associated with a history of healthcare renouncement ﻿

	OR	95 % CI	*p*
Sociodemographic profile			
Being Haitian			
No	1		
Yes	1.25	0.72–2.17	0.42
Being in France, less than a year:			
No	1		
Yes	1.32	0.62–2.81	0.47
Non applicable	1.46	0.49–4.33	0.50
Social vulnerability			
No	1		
Yes	1.55	0.47–5.14	0.48
Marital status			
In a couple/family/roomate	1		
Single	1.52	0.91–2.54	0.11
Having financial difficulties			
No	1		
Yes	1.81	1.01–3.23	**0.05**
Having financial help			
No	1		
Yes	0.94	0.56–1.57	0.81
Taking Holidays			
No	1		
Yes	1.56	0.78–3.09	0.21
Reason for coming			
Health insurance	1.74	0.80–3.79	0.17
Other	1		
Being shy to ask certain questions or to go through medical or administrative appointments			
No	1		
Yes	8.72	5.15–14.76	**<0.001**
Health profile			
Health status 3 years ago:			
The same	1		
Better	0.67	0.42–1.06	0.09
Worse	1.52	0.76–3.04	0.23
Having received dental care in the past 3 years			
No	1		
Yes	0.47	0.29–0.77	**0.003**
Having good sight			
No	1		
Yes	0.71	0.42–1.20	0.21
Medical practice, attitudes			
Having consulted the emergency ward in the past year			
No	1		
Yes	1.83	1.12–2.97	**0.02**
Having a family physician?			
No	1		
Yes	0.33	0.20–0.53	**<0.001**
Having time to explain problems when you see a doctor			
Yes	1		
No	1.72	0.87–3.40	0.30
Never consulted	2.52	0.43–14.73	0.12
Having been refused health care by a doctor in French Guiana			
No	1		
Yes	2.60	1.29–5.23	**0.01**
Never consulted	0.14	0.02–1.22	0.08
Having been refused health care by the PASS			
No	1		
Yes	1.60	0.61–4.24	0.12
Never consulted	0.68	0.42–1.10	0.34
Attitudes, practices and knowledge on health matters			
Knowing about complementary insurance			
Yes	1		
No	1.57	0.95–2.57	0.08
Knowing about about the difference between CMU and AME			
Yes	1		
No	1.17	0.70–1.95	0.54
Thinking need to feel pain or malaise to be sick			
No	1		
Yes	1.48	0.89–2.45	0.13
Knowing about infrastructures or associations which help people in administrative process			
Yes	1		
No	1.51	0.91–2.51	0.11
HIV-knowledge Quizz			
< 7 true responses	1		
7true responses	0.78	0.47–1.28	0.32
Health insurance			
Having documentation proving health insurance			
No	1		
Yes	0.49	0.23–0.65	**0.02**
Understanding mail from the health insurance			
Yes	1		
No	3.33	1.31–8.45	**0.01**
Never received	0.91	0.47–1.75	0.77

Significant variables are written in bold

Table 4 shows the global model using the significant variables from each thematic logistic regression model. Among all the variables cited below, only being embarrassed to ask certain questions or to go through medical or administrative appointments, having a family physician and having been refused care by a doctor were predictive of renouncement of care.

**Table 4:** Logistic regression. Logistic regression model using the significant variables from each thematic logistic regression model ﻿

Variables	OR	95 % CI	*p*
Having financial difficulties			
No	1		
Yes	1. 66	0.97–2.86	0.07
Being shy to ask certain questions or to go through medical or administrative appointments			
No	1		
Yes	6.81	3.98–11.65	**<0.001**
Having received dental care in the past 3 years			
No	1		
Yes	0.87	0.49–1.56	0.64
Having consulted the emergency ward in the past year			
No	1		
Yes	1.38	0.80–2.40	0.25
Having a family physician			
No	1		
Yes	0.43	0.24–0.79	**0.006**
Having been refused health care by a doctor in French Guiana			
No	1		
Yes	3.08	1.43–6.65	**0.004**
Never consulted	0.23	0.05–1.19	0.08
Having documentation proving health insurance			
No	1		
Yes	0.61	0.30–1.23	0.17
Understanding mail from the health insurance			
Yes	1		
No	2.05	0.60–6.95	0.25
Never received	0.54	0.23–1.25	0.15

Significant variables are written in bold

**Fig. 2:**
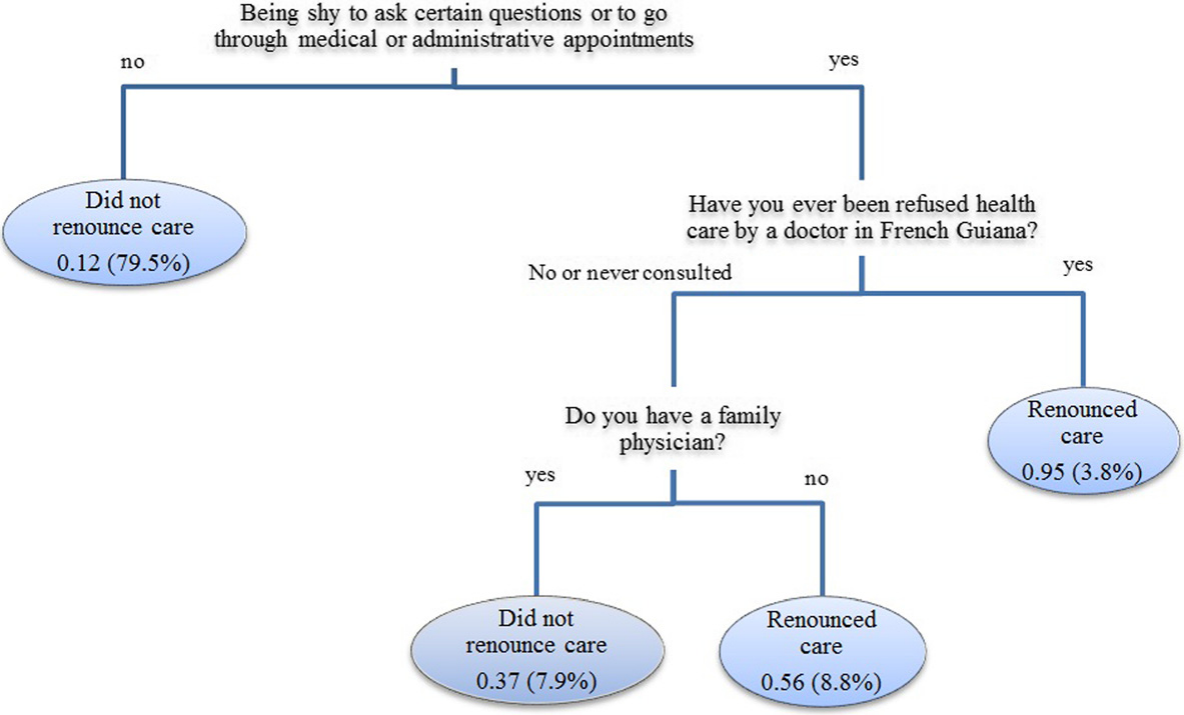
CART analysis. CART analysis for variables significantly associated with healthcare renouncement

Figure 2 shows that CART analysis identified feeling shy to ask certain questions or to go through medical or administrative appointments. CART analysis showed the two paths associated with renouncement of health care. The most salient one was feeling shy to ask certain questions or to go through medical or administrative appointments. The number shows the probability of healthcare renouncement and the one in brackets the proportion of observations in the leaf.

## Discussion

Over 70 % of the surveyed population, a vulnerable subset of the general population in Cayenne, declared it was easy to see a doctor in Cayenne. This was somewhat at odds with perceptions based on anecdotal reports. The context of health care setting at the time of the survey may have inflated the perception of the ease of contacting a doctor. In addition, the population showing up at the red cross bus may have represented a biased selection of persons least likely to renounce care thus leading to an underestimation of the main outcome. However, one in five persons had previously renounced care. In comparison, in France 15.4 % of persons declared having renounced to care in 2008, and among those earning less than 870 Euros per month this proportion reached 24 % [16]. The association between low socio economic status and access to care is often observed but the proximal forces operating are not always explicit. The present study may offer some insights on perceptions or behaviors that may be of operational interest. A number of variables were independent predictors of renouncing care. In addition, the use of CART analysis was, to our knowledge, an original way to partition the population in homogenous groups regarding the main outcome. The most salient predictor was declaring feeling shy to ask certain questions or to go through medical or administrative appointments. Having previously been refused medical care was associated with increased likelihood of having renounced care. Having a family physician was associated with a lower probability of having renounced care. The above findings suggest that factors such as self esteem or knowledge of one’s right to healthcare are possible determinants to target in order to access to care. Some persons being denied care may have generalized this experience to the health system as a whole either as a reluctance to be rejected again or as a belief that they were not entitled for health care [17]. Most of these persons were immigrants from countries where the authority and status of health professionals or administrative officers may have required the patients to be more submissive to authority. It may thus have taken some adjustment to be aware of one’s rights and to learn how to navigate the health system [18], as suggested by the fact that the most recent immigrants tended to renounce more frequently. A recent public health law suppressed the mandatory fee that patients without full coverage had to disburse. The future impact of this measure on renouncement of care is debatable but the present results suggest it may improve access.

Although, prior to the study variables such as fluency in French, transport means, employment, health insurance, residence permits were suspected determinants reported in the literature [19, 20], multivariate analyses did not retain them as significantly associated with the outcome. Perhaps in this urban area, there are a number of geographically accessible alternative offers of health care that buffer some of the difficulties of vulnerable patients. However, beyond a simple contact with a physician, follow up may have been a more difficult problem than acute care for vulnerable patients, but we did not investigate this question. Given the broad cultural diversity in French Guiana, we predicted to find that some cultural differences would arise. Although Haitian nationals seemed to renounce more often, after multivariate analyses, nationality, a proxy to culture, was not retained, suggesting that socioeconomic determinants were the confounding variable behind specific “cultural” determinants.

Caution should be exercised when making inference from the results to all poor populations of Cayenne because those that never showed up at the Red Cross truck despite the health mediators’ efforts may have represented a different subgroup less concerned by its health or unable to be present when the surveyors were on site. In addition, the surveyed areas were particularly known as poverty pockets but a number of other vulnerable persons live in other parts of town where the mix of determinants of renouncement may be different. However, and this was a deliberate choice, the surveyed population was globally very vulnerable and thus its attitudes, behaviors and practices are precious knowledge for health care actors in order to improve access to care in this population.

These results concern vulnerable populations living in a country with a universal health care system that is often presented as a model, but still struggles to achieve one of its priorities: to ensure that everyone has equal access to care. Therefore the present results may be very different from, and not applicable to, situations where persons do not have these access to health rights. As observed elsewhere, financial difficulties were linked to poverty [1]. However, here it seemed “shyness” to interact with the system and prior refusal of care stressed that, beyond the perception of one’s right to health care and perhaps one’s status in society may be even more important psychosocial determinants [2] that presumably apply beyond the particular context of French Guiana.

## Conclusion

Although these vulnerable populations reported that access to a physician was easy, 21 % of the population declared having previously renounced health care. Shyness to ask and prior refusal of care were negatively associated with access to care whereas having a regular physician seemed like facilitating access to care after adjustment for confounding. Ensuring that all public servants remain cordial to make the health system more welcoming and familiar to vulnerable groups thus seems to be important to improve access to care in Cayenne. Health mediation, may also be an important response to reduce renouncement of health care [21].
